# Examining Diabetes Management Apps Recommended From a Google Search: Content Analysis

**DOI:** 10.2196/11848

**Published:** 2019-01-16

**Authors:** Geronimo Jimenez, Elaine Lum, Josip Car

**Affiliations:** 1 Centre for Population Health Sciences Lee Kong Chian School of Medicine Nanyang Technological University Singapore Singapore; 2 Institute of Health and Biomedical Innovation Queensland University of Technology Brisbane Australia; 3 School of Clinical Sciences Faculty of Health Queensland University of Technology Brisbane Australia; 4 Global eHealth Unit, Department of Primary Care and Public Health School of Public Health Imperial College London London United Kingdom

**Keywords:** chronic diseases, diabetes, Google, health apps, mobile phone

## Abstract

**Background:**

The availability of smartphone health apps empowers people to manage their own health. Currently, there are over 300,000 health apps available in the market targeting a variety of user needs from weight loss to management of chronic conditions, with diabetes being the most commonly targeted condition. To date, health apps largely fall outside government regulation, and there are no official guidelines to help clinicians and patients in app selection. Patients commonly resort to the internet for suggestions on which diabetes app to use.

**Objective:**

The objective of this study was to investigate apps identified through a Google search and characterize these apps in terms of features that support diabetes management.

**Methods:**

We performed a Google search for the “best diabetes apps 2017” and explored the first 4 search results. We identified and compiled a list of the apps recommended in the returned search results, which were Web articles. Information about each app was extracted from the papers and corresponding app store descriptions. We examined the apps for the following diabetes management features: medication management, blood glucose self-management, physical activity, diet and nutrition, and weight management.

**Results:**

Overall, 26 apps were recommended in 4 papers. One app was listed in all 4 papers, and 3 apps appeared on 3 of the 4 lists. Apart from one paper, there were no explicit criteria to justify or explain the selection of apps. We found a wide variation in the type and the number of diabetes management features in the recommended apps. Five apps required payment to be used. Two-thirds of the apps had blood glucose management features, and less than half had medication management features. The most prevalent app features were nutrition or diet-related (19/24, 79%) and physical activity tracking (14/24, 58%).

**Conclusions:**

The ambiguity of app selection and the wide variability in key features of the apps recommended for diabetes management may pose difficulties for patients when selecting the most appropriate app. It is critical to involve patients, clinicians, relevant professional bodies, and policy makers to define the key features an app should have for it to be classified as a “diabetes management” app. The lessons learned here may be extrapolated for the development and recommendation of apps for the management of other chronic conditions.

## Introduction

We live in a digital era, where people turn first to the internet rather than a health provider to learn about a health condition or to look up health-related information [1]. Additionally, the use of health apps is increasingly widespread with over 318,000 such apps available in the market [2]. Notably, of all medical conditions, diabetes is the most commonly targeted condition by health app developers [3].

The availability of smartphone health apps may empower people even further, as these provide an opportunity to assist or support patients to better self-manage long-term conditions, like diabetes, and influence healthier lifestyles [2]. Evidence-based diabetes guidelines emphasize lifestyle management like healthy eating and physical activity to manage diabetes [4,5]. Patients who actively engage in their own care between clinic visits are more successful in managing their diabetes [6,7], by, for example, using apps with blood glucose diary features and insulin calculators [8,9].

Most diabetes apps are neither regulated nor accredited by relevant governmental bodies [10,11]. To date, no clinical guidelines for diabetes management explicitly recommend the use of specific diabetes apps, nor list mandatory or desirable features of such apps [11]. Hence, clinicians have largely been concerned about app safety and reticent about recommending diabetes management apps to patients to support self- management between clinic visits [12,13].

In the absence of any official guidance, patients exploring the use of health apps to manage diabetes often resort to the internet for suggestions. Given that in terms of search engines, Google currently holds the majority market share of >86% [14], it is likely that our hypothetical patient would “google it.” Google Trends for the search term “diabetes apps” showed increasing interest from August 2008, which peaked in August 2012 and remains at relatively high levels today [15]. We wondered about the results that such a Google search would yield and the features that the recommended apps will have.

Therefore, we aimed to investigate the characteristics (main features and type of support provided) of the apps recommended in the most popular results returned through a Google search for diabetes apps.

## Methods

### Google Search

We performed a Google search on April 18, 2018, with the following phrase: “best diabetes apps 2017.” From this search, we identified and accessed the weblinks of the first 4 search results, in line with digital market research that showed a drop in user click-through after the fifth search result [16].

### App Identification, Data Extraction, and Assessment

We identified the listed apps from each paper and extracted the following information from the papers and the Apple App store or Google Play store descriptions (respective to the listed app): App name, developer, user rating, number of ratings, whether apps were free or paid, requirements related to external blood glucose monitoring devices, diabetes management features, and general characteristics derived from paper and store descriptions.

Health interventions important for the successful management and treatment of diabetes include medications, self-management of blood glucose levels, physical activity, healthy eating, and weight management [4,5]. The presence or absence of the following app functions was noted. They were as follows: medication management—the capability to log or track medications taken or used, including insulin, other insulin-related features such as bolus calculation; self-management of blood glucose—the capability to log or track; physical activity—capability to log or track; diet and nutrition—the capability to log or track food consumption, carbohydrate, or calorie counting; and weight management—the capability to log or track weight, body mass index calculation.

Other functions were extracted as “additional functions,” which included the following: blood pressure management; glycated hemoglobin (HbA_1c_) tracking, prediction, or calculation; cholesterol management; provision of statistics and data visualization; data sharing; and the capability to connect to family members or friends, community, or health providers.

### Data Reporting and Analysis

A narrative synthesis was used to describe the papers and app store descriptions of the apps. In terms of the papers that provided the lists of recommended apps, a descriptive summary of the paper content, recommended apps, and readership of the respective website are presented. Data about the apps are presented as quantitative aggregations and tallies in terms of which and how many diabetes management features were found.

## Results

### Content Summary of Identified Papers

**Figure 1:**
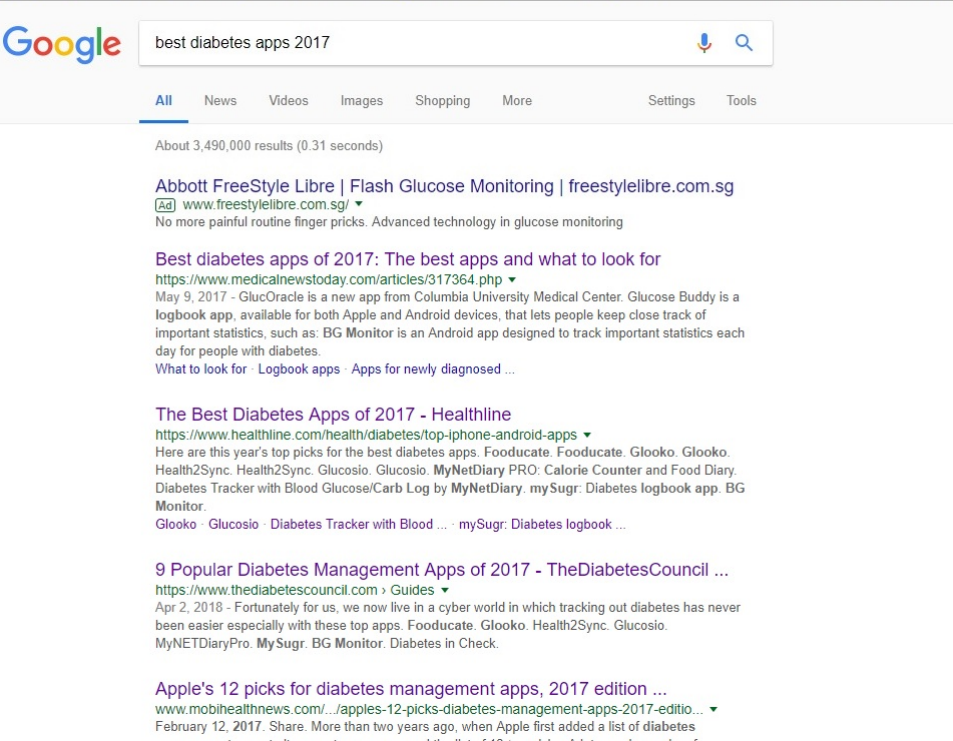
A screenshot of Google search for “best diabetes apps 2017.” Source: Google.com; screenshot taken by Geronimo Jimenez on April 20, 2018.

The first 4 search results were all papers providing lists with the “best” or “most popular” diabetes or diabetes management apps (Figure 1).

The content of each paper is described below:

1. Medical News Today—“Best diabetes apps of 2017: The best apps and what to look for” [17]. This paper guides the reader on what to look for in a diabetes management app, mentioning that it should be able to monitor blood sugar levels, carbohydrate intake, and weight management. It lists the different categories of apps (logbook, calorie counters, diet apps, carbohydrate counting apps, and general diabetes management) and reviews some of the “best diabetes apps of the year.” The website claims to have “more than 15 million monthly visits, 13 million monthly unique visitors, and 20 million monthly page views” [18].
2. Healthline.com—“The Best Diabetes Apps of 2017” [19]. This paper provides a description of the complications of diabetes and mentions the aspects that help a person live better with diabetes, such as healthy eating, exercising, taking medicines, and sticking to treatment plans. The paper then goes on to mention that several apps have emerged to help patients “keep all the pieces of their care together,” followed by a list of “this year’s top picks.” This is the only paper that explicitly mentioned app selection criteria: “We’ve selected these apps based on their quality, user reviews, and overall reliability as a source of support for people living with diabetes.” The website claims that over 85 million people access it every month [20].
3. The Diabetes Council—“9 Popular Diabetes Apps of 2017” [21]. This paper describes the complications related to diabetes and then mentions that “we live now in a cyber-world in which tracking diabetes has never been easier especially with these top apps.” The site claims to be the “#1 diabetes blog reaching millions of readers” [22]. We contacted the organization for more accurate readership figures but received no reply.
4. MobiHealthNews—“Apple’s 12 picks for diabetes management apps, 2017 edition” [23]. This paper reports on Apple’s list of 12 diabetes management apps for 2017. To ascertain Apple’s selection criteria for this list, we contacted the author of the paper directly. The author mentioned that at times, the Apple Store would publish “best apps” lists for different apps categories or purposes but, unfortunately, was not able to shed any light on the criteria Apple used to come up with the list. The site claims to reach “>over 150,000 readers every month” and that its readers are “senior-level decision makers and influencers in healthcare and technology companies” [24].

**Table 1:** Diabetes management-related functions included in each app (n=24).

App Name^a^	Medication management	Blood glucose management	Physical activity features	Nutrition or diet features	Weight management	Additional useful features
Accu-Check Connect Diabetes Management App	Yes	Yes	Yes	Yes	Yes	Data sharing and blood pressure monitoring
BeatO	N/A^b^	Yes	Yes	Yes	N/A	Diabetes education
BG Monitor	Yes	Yes	Yes	Yes	N/A	Statistics and data visualization
Blip Notes, aka Tidepool Mobile	N/A	Yes	Yes	Yes	N/A	Aggregates data from different devices
Calorie Counter & Diet Tracker by MyFitnessPal	N/A	N/A	Yes	Yes	N/A	Connects to communities
Carb counting with Lenny	N/A	N/A	N/A	Yes	N/A	Food guide
Dexcom Follow	N/A	N/A	N/A	N/A	N/A	App for recipient of shared data
Dexcom G5 Mobile	N/A	Yes	N/A	N/A	N/A	For continuous glucose monitoring
Diabetes Kit Blood Glucose Logbook	Yes	Yes	Yes	Yes	Yes	HbA_1c_^c^ monitoring, data sharing, and blood pressure monitoring
Diabetes Tracker with Blood Glucose or Carb Log by MyNetDiary	Yes	Yes	Yes	Yes	N/A	Analysis and charts
Diabetes:M	Yes	Yes	N/A	Yes	N/A	Data export options
DiabetesConnect	Yes	Yes	N/A	Yes	N/A	Data export options, features can be turned off
Fooducate	N/A	N/A	Yes	Yes	Yes	Additionally tracks sleep schedule and moods
Glooko	Yes	Yes	Yes	Yes	N/A	Statistics and data visualization as well as data export options
GlucOracle	N/A	N/A	N/A	Yes	N/A	Projects blood glucose levels based on meal
Glucose Buddy	Yes	Yes	Yes	Yes	Yes	HbA_1c_ and blood pressure functions, data export options
Glucosio	N/A	Yes	N/A	N/A	Yes	HbA_1c_, cholesterol, blood pressure, and ketones monitoring functions
Health2Sync	N/A	Yes	N/A	N/A	Yes	Blood pressure monitoring, connects with family and friends, and data exporting options
Helparound - diabetes dialysis	N/A	N/A	N/A	N/A	N/A	Information and resources for conditions and connects to community for help and support
Lose It!–Calorie Counter	N/A	N/A	Yes	Yes	Yes	Allows input of fitness goals
MyNETDiaryPro	N/A	N/A	Yes	Yes	Yes	Can sync with devices for other features and connects with friends and dietitian
mySugr: Diabetes Tracker Log	Yes	Yes	N/A	Yes	N/A	Coaching services with the upgrade
One Drop for Diabetes Management	Yes	Yes	Yes	Yes	N/A	Can connect to experts
OneTouch Reveal	Yes	Yes	Yes	Yes	N/A	Data visualization and data export options
Total functions, n (%)	11 (46)	16 (67)	14 (58)	19 (79)	8 (33)	N/A

^a^Two apps were excluded from the analysis (Diabetes in Check and GlucoSuccess) because these were no longer available in the app store.

^b^N/A: not available.

^c^HbA_1c_: glycated hemoglobin.

### Identified Apps and Corresponding Characteristics

A total of 26 apps (after removing duplicates) were identified (Multimedia Appendix 1). Of these, 2 apps were no longer available or were removed from the app stores (GlucoSuccess and Diabetes in Check) and were hence excluded from the analysis. One app was listed in all 4 papers (mySugr), and 3 apps appeared on 3 of the 4 lists (BG Monitor, Glooko, and myNetDiaryPro). In addition, 20 apps had a user rating, which varied widely among apps; 14 had user ratings of ≥4.5 (out of 5), 5 apps scored ratings <3.0, and the remaining app rated 3.3. Likewise, the number of ratings per app varied greatly; 2 apps received 384,000 and 88,000 ratings, respectively, whereas 4 apps had >4500 ratings. On the other end, 8 apps received between 100 and 700 ratings, 5 apps received 10-99 ratings, and 1 app received fewer than 10 ratings.

While 10 apps were completely free to use, 11 were either free to access but offered in-app purchases to unlock additional features, or had both a free and a paid version with more features. Three apps required payment or a subscription to access the app, although one of them (Glooko) would be free if sponsored by a doctor or covered by health insurance.

Three of the apps were part of a package that entailed purchasing a blood glucose monitoring device to use the app, that is, the Dexcom G5 Mobile Continuous Monitoring System and Roche’s Accu-Chek glucose meter. Therefore, although the app was free, users do have a financial outlay upfront. In addition, another app (One Touch Reveal) was part of a package with a glucometer, but the app can still be used without the device. Similarly, 8 other apps can be connected to blood glucose monitoring devices but would still function without a specific device. Overall, 5 apps required upfront payment either as a subscription to the app or the purchase of a required blood glucose monitoring device.

We observed wide variability among the analyzed apps in terms of features and functions for diabetes management (Table 1). Two-thirds (16/24, 67%) had blood glucose management features, and less than half (11/24, 46%) had medication management features. The most prevalent feature was a nutrition or diet function present in 79% of the apps (19/24). In addition, 58% (14/24) included functions for physical activity tracking, and 33% (8/24) had weight tracking functions. Additional useful functionalities available in the apps were statistics and data visualization (eg, charts and graphs), data sharing options, the capability to connect to a community, friends and family, or health provider or expert or dietician. Some apps included additional monitoring functions for blood pressure, HbA_1c_, and cholesterol.

## Discussion

### Principal Findings

This study explored the features of the “best/top diabetes management apps of 2017” recommended by the first 4 papers returned through a Google search. We found wide variation in the type of apps being recommended and in the number of diabetes management features included, reflecting ambiguous interpretation of the “diabetes management” concept.

The wide variability among selected apps signals no consensus regarding what diabetes management means. For instance, although there is no doubt that physical activity and healthy eating habits are important for managing diabetes, considering apps that *only* provide these features, “diabetes management” apps remains unclear and may be misleading. Several of these recommended apps only focused on diet and nutrition tracking and physical exercise which, while important, may not in itself be sufficient to manage diabetes. In addition, the selection of apps for these lists suggests little or no input from diabetes specialists (eg, diabetologists, diabetes nurse educators, and so forth) who would be mindful of *all* aspects required to manage this condition.

Are these apps capable of supporting the management of diabetes as the papers claimed? It depends. If we consider the capability of an app to log and track blood glucose levels to be an essential element for the management of diabetes, as defined by evidence-based diabetes guidelines [4,5], then 16 of the 24 available apps could be recommended for the management of diabetes based simply on the availability of this function (without exploring it in further detail). However, if we determine that the app must also support medication management, the number of apps deemed suitable decreases to 11. These results correlate with studies that found that, although diabetes management apps may have relatively high scoring functionality and aesthetics aspects, the majority do not integrate the most important diabetes management tasks [25]. Should apps that target one or two aspects of diabetes management be considered “diabetes management” apps? If so, which aspect or aspects should be deemed essential? The fact that these articles recommended some single-feature apps under the banner of “diabetes management” apps may mislead users about what to expect regarding the health consequences of living with diabetes and its successful management.

Taking all of the above into consideration, we may need to take a collective step back to examine the following questions: (1) what is diabetes management and (2) what does it really entail for each part of the patient journey? Conceptual segmentation can be helpful in designing or guiding the selection of diabetes apps—for example, in terms of the stage of diagnosis (newly diagnosed and delayed diagnosis), which health goals to emphasize at various stages, patient education or information about diabetes and health consequences, and types of support required (clinical, social or community, practical help).

For clinicians, these findings mean that patients with diabetes may be or become confused about what it means to have diabetes and how to manage it. Apps focusing solely on healthy eating and physical activity recommended under the “banner” of diabetes apps may mislead patients into thinking these strategies alone are adequate to keep diabetes in check. As such, there is a key role for clinicians to guide not only their patients but also the people creating these potentially very influential lists. Similarly, for policy makers involved with enacting health-related policies and legislation, these findings emphasize a need to work with professional organizations (eg, American Diabetes Association and American Association of Diabetes Educators) to establish and clearly define what diabetes management should “look like” in a health app based on the existing diabetes management guidelines. Jointly endorsed guidance from policy makers and professional organizations on essential features of a diabetes management app would raise not only the quality bar for app development but also promote patient safety.

More transparency of app selection for recommendations is needed, as there were no explicit criteria to justify or explain the selection of apps, apart from the Healthline.com paper [19]. Other studies have also pointed out that search capabilities in app stores are limited and algorithms are not transparent, which increases the difficulty for patients to find the most suitable app to manage their condition [10]. The criteria should be clearly stated so that users know why an app made the cut for a certain list. Given the high potential of such lists in influencing patient selection of apps, nontransparency of app selection is concerning; this will also assure users that apps were not listed because of industry influences or other unclear reasons.

For app recommendations to be user-friendly, we need a more nuanced provision of recommendations tailored to an individual patient’s journey with diabetes. For example, apps with physical activity and healthy nutrition functions may be sufficient for patients recently diagnosed with type 2 diabetes, whereas those being treated with medications and insulin may require blood glucose and medication management functions. Different types of apps may be more effective for type 1 or type 2 diabetes, younger patients, older adults, and so forth. The MedicalNewsToday article [17], for example, provided the features of their recommended apps and categorized the list into logbook apps, calorie or carbohydrate counters, diet apps, and general diabetes management apps, which may provide better guidance for users.

### Limitations

This study has several limitations. First, Google searches vary depending on the country location in which they were performed. Therefore, this same search performed elsewhere may result in different retrieved websites. However, we investigated the reach and readership of each of the websites we accessed, in terms of the number of visitors and readers, which was substantial. Second, given the rapid changes in this field, some apps may no longer be available in the app stores. For example, at the time of analysis, 2 out of the 26 recommended apps were no longer available. Nonetheless, to the best of our knowledge, the rest of the apps included in this study are still widely used and available.

Although the focal point of this study was on diabetes apps, these findings and implications also apply to health apps for the management of other chronic conditions. It is important to clearly define what the management of a chronic condition entails to inform the development of safe and efficient apps that may help in managing such conditions. For this to happen, it is essential for patients, clinicians, professional bodies, and policy makers to interact and be involved with app developers, so that apps are appropriately designed to tackle key aspects of the target condition. Subsequently, these requirements should be made known to the wider public, so that organizations and websites promoting apps for the management of a chronic condition are aware of the required app features and functions.

### Conclusions

The ambiguity of app selection and the wide variability in key features of the apps recommended are largely not helpful in guiding patients to select the most appropriate diabetes management apps. It is imperative for websites and organizations to be as transparent as possible when recommending apps, by clearly stating the selection criteria and justifications for the recommendations. There is a clear need to involve patients, clinicians, relevant professional bodies, and policy makers to define what makes an app a “diabetes management” app, including specifying the minimum features an app should have to be classified in this category. The lessons highlighted in this study could be extrapolated to other chronic diseases to inform the development and recommendation of safe and reliable apps.

## Appendix

Multimedia Appendix 1 List of recommended apps and characteristics as described in articles and app store descriptions.

## Abbreviations

HbA_1c_: glycated hemoglobin
