# Supplementary material for: Examining Diabetes Management Apps Recommended From a Google Search: Content Analysis
Source: JMIR Mhealth Uhealth. 2019 Jan 16;7(1):e11848. doi: 10.2196/11848 (PMC6352016; doi:10.2196/11848)
Supplement: Multimedia Appendix 1 [file mhealth_v7i1e11848_app1.pdf]

Multimedia Appendix 1: List of recommended apps and characteristics as described in articles and app store descriptions

App name	Developer	Article(s) reccomending?[a]	Store availability[b]	User rating & Number of ratings	Free/ paid / in-app purchases	External device requirements (e.g. glucometer)	General characteristics as described in articles and app store descriptions
Accu-Check Connect Diabetes Management App	Roche Diabetes Care	MHN	AAS / GPS	2.1/5 101 ratings	Free	Requires device to access app	Companion app for Roche's Accu-Check glucometers, takes in data from wireless glucometers and includes insulin dose calculator for users with a prescription.
BeatO	Health Arx Technologies	DC, HL	AAS / GPS	4.5/5 8 ratings	Free	Connects to devices, but app still functional without a device	App helps keep track of high and low glucose levels and what foods to eat. BeatO has tools to help manage high and low glucose levels. Provides basic educational material about food, and concerns users may have (e.g. things you should know about tattooing and diabetes). Can sync with daily devices including Fitbit.
BG Monitor	Gordon Wong	DC, HL, MNT	GPS	4.5/5 637 ratings	Free and in-app purchases	No external device requirements (need to check)	Monitors blood glucose and insulin. It calculates carbohydrates and supports U.S. and international units of glucose measurements. Takes photos of meals, creates CSV file reports and backs up data to Google Drive to ensure "you always have vital information to share with your healthcare team".
Blip Notes, aka Tidepool Mobile	Tidepool	MHN	AAS / GPS	No ratings yet	Free	Linked to a website, which uses other devices (not sure if requires other devices)	Platform for aggregating data from different diabetes devices. App allows the user to take qualitative notes about a reading on a particular device, and supports hashtags to connect readings that happen with a regular event e.g. meal or sports practice.
Calorie Counter & Diet Tracker by MyFitnessPal	MyFitnessPal.com	MHN, MNT	AAS / GPS	4.7/5 384,400 ratings	Free and in-app purchases	No external device requirements	Well-known app for tracking diet and exercise. It includes a comprehensive food database and connects with more than 50 apps and devices for tracking fitness; includes a built-in activity tracker. Calorie counter app designed to help users lose weight. It has more than 6 million foods in its database, and is equipped with a barcode scanner. All nutrients can be tracked, and the app allows users to set goals. Also allows users to log exercise and steps and find out how many calories they are burning. Users can connect with friends.
Carb counting with Lenny	Medtronic, Inc.	MNT	AAS / GPS	2.5/5 12 ratings	Free	No external device requirements	Designed to help ease children or adults new to diabetes into managing their conditions. Helps users monitor their carbohydrate intake. Has food guide feature to learn the carbohydrate values of different foods. A useful app to understand the carbohydrate values of various foods.
Dexcom Follow	Dexcom	MHN	AAS / GPS	2.9/5 154 ratings	Free	Requires device to access app	App is for friends and family of G5 users who can monitor their own glucose with the G5 Mobile app. Can view shared data from five different sharers.
Dexcom G5 Mobile	Dexcom	MHN	AAS / GPS	2.9/5 602 ratings	Free	Requires device to access app	Allows users of Dexcom's G5 continuous glucose monitor to view their readings and share them. Forwards trend alerts when a user's glucose level is too high or too low.
Diabetes in Check		DC, HL		Not able to access Apple App or Google Play Store description page			
Diabetes Kit Blood Glucose Logbook	Diabetes Labs, LLC	MHN	AAS	2.8/5 35 ratings	Free and in-app purchases	Connects to devices, but app still functional without a device	Users can track blood glucose, insulin dosing, food, HbA1c, blood pressure and activity. Data input is via manual entry, integration with Apple's HealthKit, and by taking a picture of the reading on the glucometer's screen. Syncs with Dexcom's continuous glucose monitors and includes an insulin calculator.
Diabetes Tracker with Blood Glucose/Carb Log by MyNetDiary	MyNetDiary Inc.	HL	AAS / GPS	4.6/5 143 ratings	Paid and in-app purchases	Connects to devices, but app still functional without a device	Built-in features allow easy and automatic recording of physical activities and foods. Monitors water intake, weight, A1c, cholesterol, and net carbohydrates. Food grades, reports on personal progress, and virtual coaching helps users better understand how to take charge of their health.
Diabetes:M	Sirma Medical	MNT	AAS / GPS	4.6/5 155	Free and in-app	Connects to devices, but	App enables users to track food intake, monitor diet, enter important values in the logbook,

	Systems JSC			ratings	purchases	app still functional without a device	analyze data quickly. Equipped with an insulin bolus calculator. Reminds users for tests or appointments. Data can be exported to a doctor.
DiabetesConnect	SquareMed Software GmbH	MNT	AAS / GPS	4.5/5 36 ratings	Free and in-app purchases	No external device requirements	Users can record blood glucose, meals, injections and medications and other important statistics directly into the app. Features can be turned off and on as needed to avoid clutter. Data can be exported to a doctor.
Fooducate	Fooducate, Ltd.	DC, HL	AAS / GPS	4.7/5 11,100 ratings	Free and in-app purchases	No external device requirements	App is not only a weight loss coach but also for monitoring carbohydrates, exercise, hunger, sleep schedule and moods. Explains pros and cons of certain foods and has features that allows users to customize needs in case of food allergies.
Glooko	Glooko, Inc.	DC, HL, MNT	AAS / GPS	3.3/5 19 ratings	Free if sponsored by doctor or covered by insurance. Subscription fee otherwise.	Connects to devices, but app still functional without a device	App can connect with most blood glucose meters to keep track of carbohydrate counts and medicine usage, diet and exercise. Allows users to set reminders for meals and medication. Handy charts to show real-time events and trends which can be shared with doctor.
GlucOracle	Sassafras Tech Collective, LLC	MNT	AAS / GPS	No ratings yet	Free	No external device requirements	App helps users manage blood glucose by forecasting blood glucose levels before the intended meal. Users also enter actual blood glucose levels after the meal. Feedback about the nutrients in meals are given to help users monitor and improve their food intake.
Glucose Buddy	Azumio Inc.	MNT	AAS / GPS	4.8/5 4,700 ratings	Free and in-app purchases	No external device requirements	Logbook app that lets user keep track of blood glucose, carbohydrate intake, medication use, activity levels, A1c, blood pressure and weight. Provides reminders and a calendar with daily readings to easily track blood glucose levels. Exports data into printable formats for doctor/healthcare provider.
Glucosio	Glucosio Foundation	DC, HL	AAS / GPS	No ratings yet	Free	No external device requirements	Tracks body weight, ketones, cholesterol, vital signs and more for people with Type 1 or Type 2 diabetes. Allows user to set alarms for medications and meal times. Can back up all information onto Google drive. Anonymously allows diabetes researchers to track records which provides information to learn more about diabetes.
GlucoSuccess	ResearchKit study from Massachusetts General Hospital	MHN		No longer available in Apple App Store			
Health2Sync	H2 Inc.	DC, HL	AAS / GPS	No ratings yet	Free and in-app purchases	No external device requirements	Can connect with friends and family, for a great motivation system. Allows user to see blood pressure and vital signs along with its own diary, including trends for what causes spikes in blood glucose and mood.
Helparound - diabetes dialysis	HelpAround Inc	MHN	AAS / GPS	4.6/5 19 ratings	Free	No external device requirements	Provides user with a local support group of nearby helpers who could be of assistance if the user (person with the condition) shares in the forum that they do not have their supplies, are feeling a bit off, and need someone to help.
Lose It! – Calorie Counter	FitNow	MHN	AAS / GPS	4.7/5 88,000 ratings	Free and in-app purchases	No external device requirements	Nutrition tracking/ weight loss app that helps user track their diet and exercise, and set and meet fitness goals. Allows user to upload a food item just by taking a picture of it.
MyNETDiary Pro	MyNetDiary Inc.	DC, HL, MNT	AAS / GPS	4.5/5 458 ratings	Paid and in-app purchases	Connects to devices, but app still functional without a device	Personal diary for tracking your food intake, weight loss goals, daily exercise. Can sync this app with your iOS Health app. Claims that users can track anything via customised trackers in the apps. Allows connecting with a registered dietitian who can help you with questions you may have or when you need the extra support. Helps users get a better understanding of foods — has its own database of over 700,000 different foods.

mySugr: Diabetes Tracker Log	mySugr GmbH	MHN, DC, HL, MNT	AAS / GPS	4.6/5 584 ratings	Free and in-app purchases	Connects to devices, but app still functional without a device	App helps Type 1 and Type 2 diabetes patients to track their food intake and blood glucose levels. The pro version adds coaching services and a bolus calculator, as well as reminders. App is registered with the FDA as a Class 1 device. Has different levels depending on what you purchase. Can sync with other devices. Also acts like a personal coach to achieve your personal goals and stay on target. Pro (paid) version gives more information on blood sugar reminders and meals. Can create PDF or Excel files to share results with doctor. A personal certified diabetes educator is provided upon further upgrade (pay more).
One Drop for Diabetes Managemen t	Informed Data Systems, Inc.	MHN	AAS / GPS	4.5/5 4,500 ratings	Free and paid versions	Connects with several devices, but app still functional without a device	An FDA-approved app, has both a free and a pro version. On the companion app, users can log a variety of information: glucose, diet, activity and insulin. Users can share that information anonymously with a community of users, and the platform also offers users actionable insights based on their data.
OneTouch Reveal	LifeScan Inc.	MHN	AAS / GPS	4.7/5 12,400 ratings	Free	Connects with device, but app still functional without a device	This companion app receives meter readings wirelessly, and users can use it to see current data and colourful summary charts. Users can also share information with healthcare professionals via email or text.

[a] DC = Diabetes Council; HL = Health Line; MNT = Medical News Today; MHN = MobiHealthNews

[b] AP = Apple App Store; GP = Google Play Store
